# Characterization of a novel *Entamoeba histolytica* strain from Burkina Faso, Africa, possessing a unique hexokinase-2 gene

**DOI:** 10.1051/parasite/2011184287

**Published:** 2011-11-15

**Authors:** J. Suzuki, S. Kobayashi, M. Imada, M.E. Tolba, T. Takeuchi

**Affiliations:** 1 Division of Clinical Microbiology, Department of Microbiology, Tokyo Metropolitan Institute of Public Health Tokyo Japan; 2 Department of Tropical Medicine and Parasitology, School of Medicine, Keio University Tokyo Japan; 3 Department of Medical Genome Science, Graduate School of Frontier Sciences, University of Tokyo Tokyo Japan

**Keywords:** *Entamoeba histolytica*, isoenzyme, hexokinase, genetic variation, isoelectric focusing, *Entamoeba histolytica*, isoenzyme, hexokinase, variation génétique, concentration isoélectrique

## Abstract

An *Entamoeba histolytica* strain (BF-841 cl1) that originated from Burkina Faso, Africa presented with novel, polymorphic genotypes of the serine-rich *E. histolytica* protein and the anodic hexokinase-2 (HXK-2) isoenzyme band, which showed less electrophoretic mobility than that of an *E. histolytica* reference strain [HM-1:IMSS cl6 (zymodeme (Z)-II)] by starch gel electrophoresis and isoelectric focusing (IEF). The *HXK-2* gene of BF-841 cl1 had amino acid variations at four positions compared to the sequence of HM-1: IMSS cl6. These variations were absent from the sequences of four other *E. histolytica* strains with different zymodemes [KU27 (Z-II), SAW1627 (Z-IIα-), SAW755CR clB (Z-XIV), and KU2 (Z-XIX)]. The results of IEF showed no difference in the substrate specificity of HXK (HXK-1 and HXK-2) between BF-841 cl1 and the three reference *E. histolytica* strains (HM-1:IMSS cl6, SAW755 clB, and KU27). It was also confirmed that BF-841 cl1 was able to form liver abscesses in Syrian hamsters.

## Abstract (fr)

Une souche d’*Entamoeba histolytica* (BF-841 cl1), originaire du Burkina Faso en Afrique, présente un nouveau polymorphisme génétique pour la protéine riche en sérine et aussi pour l’hexokinase anodine (HXK-2) qui a une moindre mobilité électrophorétique que celle de la souche d’*E. histolytica* de référence [HM-1:IMSS cl6 (zymodème (Z)-II)] en électrophorèse sur gel d’amidon et dans l’analyse en focalisation isoélectrique (IEF). Le gène *HXK-2* de la souche BF-841 cl1 présente des variations d’acides aminés à quatre positions par rapport à la souche de HM-I:IMSS cl6. Ces variations diffèrent aussi de celles de quatre autres souches d’*E. histolytica* avec différents zymodèmes [KU27 (Z-II), SAW1627 (Z-IIα-), SAW755CR clB (Z-XIV) et KU2 (Z-XIX)]. Les résultats de l’IEF ne montrent aucune différence dans la spécificité du substrat de HXK (HXK-1 et HXK-2) entre la souche BF-841 cl1 et les trois souches référencées (HM-1:IMSS cl6, SAW755 clB et KU27). Il a aussi été confirmé que BF-841 cl1 est capable de former un abcès du foie chez des hamsters syriens.

## Introduction

The species-specific isoenzyme patterns (zymodemes) of hexokinase (HXK) and phosphoglucomutase (PGM) have been utilized for the differential identification of *Entamoeba histolytica* from morphologically indistinguishable, nonpathogenic *Entamoeba dispar* (Sargeaunt, 1988). Cathodic HXK-1 and anodic HXK-2, the two isoenzyme electrophoretic bands of *E. histolytica* HXK, usually exhibit more anodic migration than those from *E. dispar*; an exception is the HXK of an *E. histolytica* isolate, classified as zymodeme XIII (Z-XIII), which shows the same mobility as the HXK of *E. dispar* (Sargeaunt, 1988). Other variations in the mobility of HXK isoenzyme bands have been reported in *E. histolytica* strain SFL-3 (Ortner *et al.*, 1997) and in some strains of *E. histolytica*-like species from nonhuman primates (*e.g.* JSK2004 cl2; Suzuki *et al.*, 2007). In strain SFL-3, in addition to the usual HXK-1 and HXK-2 bands, an independent third HXK isoenzyme band (HXK-3) that is more cathodic than the other bands is observed. In strain JSK2004 cl2, the HXK-1 band is more cathodic than the typical HXK-1 of *E. histolytica*.

An *E. histolytica* strain (BF-841) exhibiting a novel variation in the HXK-2 band was incidentally isolated from an asymptomatic patient.

In the present study, the axenic clone (BF-841 cl1) was established and used for a molecular characterization study in which some distinct profiles were clarified. We examined the strain’s ability to induce experimental liver abscess in hamsters and the mRNA levels of the galactose/*N*-acetyl-d-galactosamine-inhibitable (Gal/GalNAc) lectin gene as indexes of BF-841 cl1 virulence.

## Materials and Methods

### Isolate of *E. histolytica*, *E. dispar*, and an *E. histolytica*-like variant

The BF-841 strain of *E. histolytica* was isolated from a Japanese patient who was infected with *E. histolytica* during a long-term stay in Burkina Faso, northwest Africa, for volunteer activity. The strain was cultured using Robinson’s xenic culture medium (Robinson, 1968). The axenic strain was established from a monoxenic culture with viable *Crithidia fasciculata* (ReF-1:PRR, ATCC No. 50083) by the classical approach (Diamond, 1983) using YIMDHA-S medium (Suzuki *et al.*, 2008). The clone of BF-841 (BF-841 cl1) was obtained by the classical method (Gillin & Diamond, 1978). *E. histolytica* [HM-1:IMSS cl6 (Z-II, axenic clone), KU27 (Z-II, axenic strain; Aleyla *et al.*, 2010), SAW1627 (Z-IIα-,axenic strain; Razmjou *et al.*, 2006), SAW755CR clB (Z-XIV), KU2 (Z-XIX, axenic strain; Haghighi *et al.*, 2002)], the *E. histolytica*-like variant (for which Tachibana *et al.*, 2007 have proposed the name *Entamoeba nuttalli*) from nonhuman primates (JSK2004 cl2, axenic clone; Suzuki *et al.*, 2007), and *E. dispar* (AS 16 IR, axenic strain; Suzuki *et al.*, 2008) were used as references. HM-1:IMSS cl6, SAW755CR clB, and xenic SAW1627 were kindly supplied by the late Dr Louis S. Diamond (National Institute of Health) and by the late Dr Peter G. Sargeaunt (London School of Hygiene and Tropical Medicine), respectively. All these strains and clones of *Entamoeba* spp. were subcultured in YIMDHA-S medium for use in the present study.

### Isoenzyme analysis

Isoenzyme analyses were performed by starch gel electrophoresis (SGE) (Sargeaunt, 1988) and isoelectric focusing (IEF) using precast Novex^®^ IEF gels (pH 3-7), Novex^®^ IEF buffer systems, and XCellTM SureLock Mini-Cells (Invitrogen Co., Carlsbad City, CA, USA) to characterize the HXKs of the six strains of *Entamoeba* spp. [*E. histolytica*: HM-1:IMSS cl6, SAW755CR clB, KU27, and BF-841 cl1; *E. dispar*: AS 16 IR; *E. histolytica*-like variant (*E. nuttalli*): JSK2004 cl2]. For IEF, samples of the examined *Entamoeba* strains were first lysed with two freeze-thaw cycles following the addition of enzyme stabilizers, EDTA, dithiothreitol, and ε-aminocaproic acid, each to a final concentration of 1 mM. Next, the samples were loaded onto precast Novex^®^ IEF gels under the conditions specified in the manufacturer’s instructions. The HXK (glucokinase and mannokinase) activities of electrophoresed isoenzymes were visualized by production of formazan dye from the NADPH formed in a coupled glucose-6-phosphate dehydrogenase (G6PDH) reaction with each of the substrate mixtures for glucokinase (Sargeaunt, 1988) and mannokinase (Jelnes, 1971). The enzymes G6PDH XV (Cat. No. G6378) for the glucokinase and mannokinase assays, phosphomannose isomerase (MPI; Cat. No. P2621) and phosphoglucose isomerase (GPI; Cat. No. P5381) for the mannokinase assay were purchased from Sigma-Aldrich Co. (St. Louis, MO, USA). Mannokinase was also visualized in the glucokinase reaction mixture by using G6PDH II (Cat. No. T-51; Asahi Kasei Pharma Co., Chiyoda-ku, Tokyo, Japan), which has 33% relative activity for lactonization from mannose-6-phosphate (M6P), as compared with that from G6P (100%). In every IEF assay, the same amoeba lysates (six strains of three *Entamoeba* spp.) were used as samples, and the same volumes were applied for the comparison of HXK activity.

### PCR and sequence analysis

The genomic DNA of BF-841 cl1 was isolated with the QIAamp^®^ DNA Mini Kit (Qiagen GmbH, Hilden, Germany). PCR amplification of the template DNA was performed using previously reported primer sets targeting the small subunit ribosomal RNA (SSU rRNA) gene (Suzuki *et al.*, 2007), the nucleotide base sequences of two protein-coding genes [chitinase and the serine-rich *E. histolytica* protein (SREHP)], and the protein-noncoding locus 1-2 genes (Haghighi *et al.*, 2002). For the genetic analysis of a pathogenicity factor of *E. histolytica*, we performed PCR amplifications using original primer sets (four sets of Eh-LecF and Eh-LecR) (Table I) targeting the Gal/GalNAc lectin gene in six *E. histolytica* strains (Petri *et al.*, 1987; Ravdin *et al.*, 1982). ExTaq DNA polymerase (Takara Bio Inc., Shiga, Japan) was used in these PCR amplifications with the following cycle conditions: Taq activation at 94 °C for 3 min; 35 cycles of denaturation at 94 °C for 40 s, annealing at 50 °C (chitinase, SREHP, and locus 1-2) or 56 °C (SSU rRNA and Gal/GalNAc lectin) for 40 s, and extension at 72 °C for 1 min; and a final extension at 72 °C for 5 min. The genes for HXK, GPI, PGM, and malic enzyme (ME), which are utilized for the zymodeme assay, were also amplified and sequenced from five strains of *E. histolytica* that possess different zymodemes. The primers for genes *HXK*, *GPI* (Tachibana *et al.*, 2007), and *PGM* (Takano *et al.*, 2009) and the original EhME-F and EhME-R primers for the *ME* gene (shown in Table I) were used for the DNA amplification. The amplified *SREHP* and *GPI* genes were cloned using the QIAGEN PCR Cloning Kit (Qiagen GmbH, Hilden, Germany). In PCR amplifications targeting the *HXK*, *GPI*, *PGM*, and *ME* genes, LATaq DNA polymerase (Takara Bio Inc.) was used with the following cycle conditions: denaturation at 95 °C for 30 s, annealing at 58 °C for 30 s, and extension at 72 °C for 1 min.

**Table I.** Oligonucleotide primers used for PCR assays targeting galactose/*N*-acetyl-d-galactosamine-inhibitable (Gal/GalNAc) lectin and malic enzyme (ME) genes of *Entamoeba histolytica*.

	Primer name		Primer sequence (5’ to 3’)	Position	Accession number
Gal/GalNAc	Eh-Lec1F	(forward)	ATTTTGGTATTATTTTATGCTTCA	7–30	XM_645442
	Eh-Lec1R	(reverse)	TGATGAAACAGATGATTTAAAG	1065–1086	XM_645442
	Eh-Lec2F	(forward)	ATCAGTAAATATGCAGGAAAAG	925–946	XM_645442
	Eh-Lec2R	(reverse)	TTATCAACATTAATACATTTTGGA	1860–1883	XM_645442
	Eh-Lec3F	(forward)	TGTATTAAAGTATCTCCATATGA	1636–1658	XM_645442
	Eh-Lec3R	(reverse)	ATACTTCCAGAAGCATCACA	2857–2876	XM_645442
	Eh-Lec4F	(forward)	ATCAGATGACTTGTTCAGATG	2681–2701	XM_645442
	Eh-Lec4R	(reverse)	TGAAATTAGCATTTGTGGCA	3598–3617	XM_645442
ME	EhME-F	(forward)	ATGGCACAATTAAAAGCAGA	1–20	NW_001914864
	EhME-R	(reverse)	TAATAGCATAAGCAAGACACT	1424–1444	NW_001914864

All PCR products from the examined *E. histolytica* strains were sequenced using the ABI Prism BigDye Terminator v3.1 Cycle Sequencing Ready Reaction Kit (Applied Biosystems, Foster City, CA, USA) and an ABI PRISM 3130 Genetic Analyzer.

### mRNA levels of *E. histolytica* adhesin

For the comparison of expression levels of adhesin (Gal/GalNAc lectin) mRNA among four *E. histolytica* strains (BF-841 cl1, HM-1:IMSS cl6, SAW1627, and KU2), real-time (RT) PCR was carried out. After three days of culture, floating amoebae and cell debris were removed by gentle decanting of the culture supernatant, and the amoebae of each strain adhering to the glass walls were used as samples for mRNA isolation. The amoebae were resuspended in fresh YIMDHA-S medium and adjusted to a concentration of 200,000 amoebae/mL.

The mRNA was prepared from the pre-extracted total RNA of each strain using the Oligotex-dT30 mRNA Purification Kit (Takara Bio Inc.). The total RNA was extracted using the RNA Isolation Kit (Macherey-Nagel GmbH, Düren, Germany) from the same 1,000 amoebae for each of the strains. Subsequently, cDNA was obtained from mRNA by the reverse transcriptase reaction, using the High Capacity RNA-to-cDNA Kit (Applied Biosystems). Meanwhile, the QIAamp® DNA Micro Kit (Qiagen GmbH) was used to extract DNA independently from the same 1,000 amoebae for each of the strains as a reference for comparison of quantities of cDNA obtained from mRNA among the four strains of *E. histolytica*. The characteristic fragment of the Gal/GalNAc lectin gene was amplified by TaqMan MGB RT-PCR from the cDNA as well as from the DNA as a reference. The primers and probes for RT-PCR were designed using the accessory software supplied by Applied Biosystems (Primer Express, version 2.0). The detailed sequences of the primers are as follows: ELecF (5’-GGAATTCAAACGAAGAAAAGGTAAAA-3’) and ELecR (5’-GCATCTACTTGATTTGGACATTTATCA- 3’). The fluorogenic probes [carboxyfluorescein *N*hydroxysuccinimide ester (FAM) and Minor Groove Binder (MGB)] were labeled as follows: 5’-FAMTGGAATGGATAAAGAGTCTAC- MGB-3’ (ELec-Probe).

The cDNA and the DNA were amplified with the primers and the probes in TaqMan Universal Master Mix (Applied Biosystems) using the ABI 7900 Fast RT-PCR System (Applied Biosystems) with the following conditions: 50 °C for 3 min, 95 °C for 10 min and 40 cycles of 95 °C for 15 s and 60 °C for 1 min. A standard curve was created for mRNA quantification by plotting the mRNA (cDNA) quantities from 250, 500, and 1,000 amoebae of BF-841 cl1 per 5 μL. The mRNA quantity (QmEH) of the reference strain of *E. histolytica* relative to that of BF-841 was represented as a proportionate value (the proportionate value of BF-841 cl1 was set as 1) to the mRNA (cDNA) quantity of BF-841 (QmBF841) by using the following formula:

$${\mathrm{Qm}}_{\mathrm{EH}}=({\mathrm{Qm}}_{\mathrm{EH}}\;\times \;Q_{\mathrm{EH}}/Q_{\mathrm{BF}841})/{\mathrm{Qm}}_{\mathrm{BF}841}$$

where Q_EH_ is the DNA quantity of an *E. histolytica* strain and Q_BF841_ is the DNA quantity of BF-841 (DNA quantity is per 1,000 amoebae).

### Captured-antigen level of adhesin

For comparing captured-antigen levels of adhesin among the same four strains of *E. histolytica* that were examined using mRNA, the *E. histolytica II* Kit (TechLab, Blacksburg, VA, USA) (Haque *et al.*, 1993) was used. The assay was conducted under the conditions specified in the manufacturer’s instructions, using the same protein content (2 μg protein) for each extract and the same number (2,000 amoebae) of strains; the amoebae were chilled for 5 min at 4 °C, and the number of amoebae of each strain in YIMDHA-S medium was counted. The number of amoebae per strain was adjusted to a concentration of 2 × 105/mL after washing with Hanks’ balanced salt solution twice by centrifugation (275 × *g*, 3 min). These amoebae were subjected to two freeze-thaw cycles in Hanks’ solution followed by the determination of protein contents by the Bradford protein assay (Bradford, 1976). The two cycles of freeze-thawed amoebae lysates were stored in convenient volumes (1.5 mL) at - 80 °C. The amoebic extracts were adjusted to protein concentrations of 2.0 μg/mL by dilution with the accessory diluent in the kit, and the captured adhesin contents were quantified in triplicate by an enzyme immunoassay at optical density OD450 nm, using the *E. histolytica II* Kit. The captured-antigen levels of adhesin of the reference strains of *E. histolytica* relative to those of BF-841 were represented as values proportionate to the antigen level of adhesin of BF-841 cl1. The protein content per 105 amoebae of each strain was also represented as a proportionate value. In these comparisons, the proportionate value of BF-841 cl1 was set as 1.

### Ability to form liver abscesses

The ability of the axenic-cultured BF-841 cl1 to form liver abscesses in Syrian hamsters was examined as described previously (Suzuki *et al.*, 2007).

## Results

**Fig. 1.**
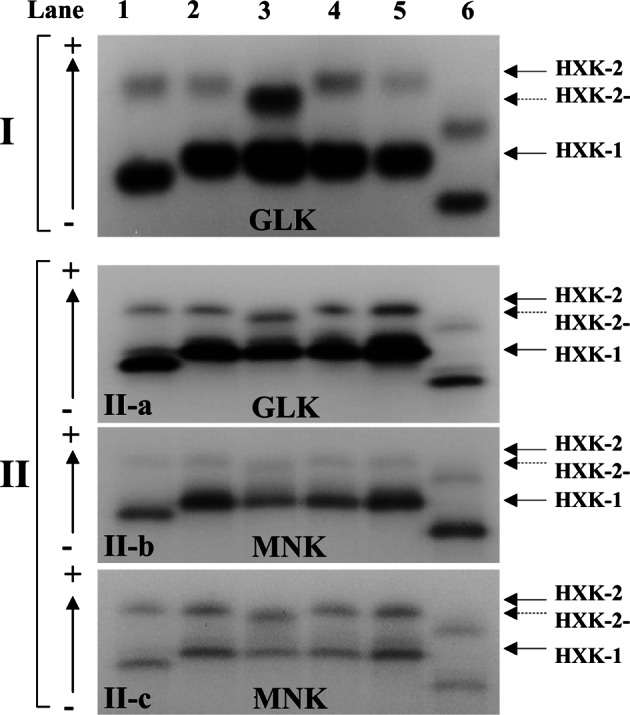
Isoenzyme patterns of hexokinase (HXK) by starch gel electrophoresis (I) and isoelectric focusing (II). Glucokinase: GLK; Mannokinase: MNK. Lane 1: JSK2004 cl2 [*Entamoeba histolytica* (Eh)-like variant (*E. nuttalli*)]; Lane 2: KU27 (Eh); Lane 3: BF-841 cl1 (Eh); Lane 4: SAW755CR clB (Eh); Lane 5: HM-1:IMSS cl6 (Eh); Lane 6: AS 16 IR (*E. dispar*).

The zymodeme of BF-841 cl1 was classified as XIV after being noted as having an HXK-2 with an unusual mobility, as observed in SGE and IEF analyses. The mobility of the anodic HXK-2 of BF- 841 cl1 was less anodic (indicated as XIVhk2-) than the mobilities of HM-1:IMSS cl6, KU27, SAW755CR clB, and JSK2004 cl2 (*E. nuttalli*), as shown by both SGE (Fig. 1: I) and IEF (Fig. 1: II). Regarding the substrate specificity of the HXKs of the six strains of *Entamoeba* spp. to phosphorylated glucose (Fig. 1: I and II-a) and mannose (Fig. 1: II-b and II-c), the reactions of HXK-1 in substrate specific assays were substantially stronger than those of HXK-2 in all six strains (Fig. 1: I, II-a, and II-b); these are the known characteristics of recombinant HXK-1 and HXK-2 of the *E. histolytica* strain (SFL-3), as reported by Kroschewski *et al.* (2000). However, when these six strains were assayed using G6PDH II directly, without MPI and GPI, no differences in mannokinase activity were observed between the isoenzymes HXK-1 and HXK-2 (Fig. 1: II-c).

The complete 1,944-bp SSU rRNA gene nucleotide sequences of BF-841 cl1 and HM-1:IMSS cl6 (Genbank accession Nos AB608092 and X65163) were identical. Typing of the genetic polymorphisms revealed that the genotypes of locus 1-2 (AB608094) and chitinase (AB608093) in BF-841 cl1 were B and C, respectively; this was previously reported by Haghighi *et al.* (2002).

**Fig. 2.**
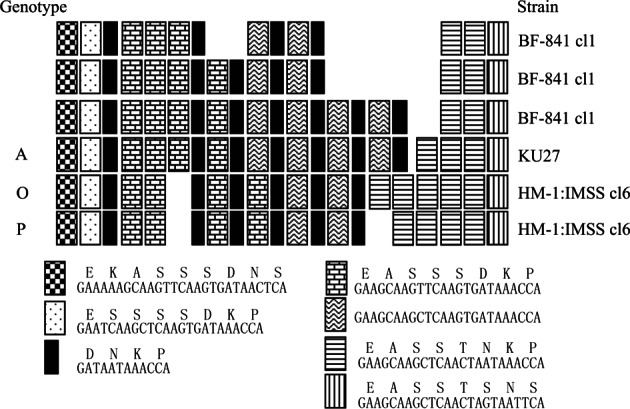
Schematic representation of polymorphisms in the repeat-containing regions of SREHP of the BF-841 cl1, KU27, and HM-1:IMSS cl6 strains of *E. histolytica*. The nucleotide (bottom) and deduced amino acid (top) sequences of the repeats are shown below.

In the PCR-amplified DNA of the *SREHP* gene of BF-841 cl1, three bands were observed (data not shown), and the DNA from each of the gel-purified PCR bands was cloned and sequenced (AB608095-7). The genotype of the most cathodic DNA band in the electrophoresed agarose gel has the highest homology with the genotype A observed in KU27; the nucleotide base sequences of the other two bands were different from all other known genotypes (Fig. 2).

The nucleotide base and amino acid sequence analyses of Gal/GalNAc lectin (heavy subunit) of BF-841 cl1 revealed that both sequences of BF-841 cl1 were identical to that the corresponding sequences in SAW755CR clB but were different from those of the four strains possessing different zymodemes (Table II).

**Table II.** Polymorphic sites of the sequences of Gal/GalNAc lectin (heavy subunit) in BF-841 cl1 and reference strains possessing four different types of zymodemes of *Entamoeba histolytica*.

		Nucleotides*			Amino acids*	
Strain	Zymodeme	1771	1810	2333	604	778
HM-1:IMSS cl6	Z-II	C	A	A	Thr	Gln
KU27	Z-II	C	A	A	Thr	Gln
SAW1627	Z-IIα-	C	A	A	Thr	Gln
SAW755CR clB	Z-XIV	A	G	G	Ala	Arg
BF-841 cll	Z-XIVhk2-	A	G	G	Ala	Arg
KU2	Z-XIX	C	G	A	Ala	Gln

* The reference sequence data of HM-1:IMSS cl6 (XM_645442) strain is represented as numbers at variable positions in the heavy subunit of Gal/GalNAc lectin.

The nucleotide base sequence homology of the HXK-1 of BF-841 cl1 (AB608087) was identical to the reference sequence of HM-1:IMSS (XM_648528), which in turn, is the same as those of SAW1627, SAW755CR clB, and KU2. However, in the sequence of the HXK-2 of BF- 841 cl1 (AB608088), eight base substitutions were present, as compared to that of HM-1:IMSS (XM_650873). The base substitutions corresponded to the four positions of substitutions in the amino acid sequence. The theoretical isoelectric point (p*I*) of HXK-2 of BF-841 cl1 was 5.19 and those of others were 5.14 (Table III). For the *GPI* gene nucleotide base and GPI amino acid sequences, two alleles (allele-1 and allele-2) have been identified in each of the representative strains with four different zymodemes [Z-II (HM-1:IMSS cl6), Z-IIα- (SAW1627), Z-XIV (SAW755CR clB), and Z-XIX (KU2); Razmjou *et al.*, 2006]. The amino acid sequence of allele-1 of BF-841 cl1 was identical to that of allele-1 of SAW755CR clB; these two strains also had the same theoretical p*I* for their GPI isoenzyme bands. However, compared to the sequence of allele-2 of SAW755CR clB, allele-2 of BF-841 cl1 contained two amino acid substitutions-glycine at position 438 and cysteine at position 449 were substituted with glutamic acid and arginine, respectively (Table IV).

**Table III.**
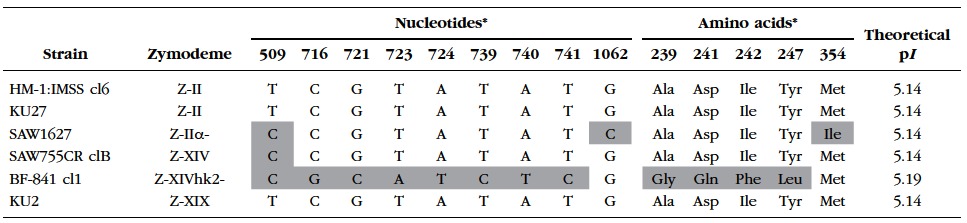
Polymorphic sites of hexokinase-2 sequences in BF-841 cl1 and reference strains possessing four different types of zymodemes of *Entamoeba histolytica*.

Strain	Zymodeme	Nucleotides*									Amino acids*					Theoretical p*I*
		509	716	721	723	724	739	740	741	1062	239	241	242	247	354	
HM-1:IMSS cl6	Z-II	T	C	G	T	A	T	A	T	G	Ala	Asp	Ile	Tyr	Met	5.14
KU27	Z-II	T	C	G	T	A	T	A	T	G	Ala	Asp	Ile	Tyr	Met	5.14
SAW1627	Z-IIα-	**C**	C	G	T	A	T	A	T	**C**	Ala	Asp	Ile	Tyr	**Ile**	5.14
SAW755CR clB	Z-XIV	**C**	C	G	T	A	T	A	T	G	Ala	Asp	Ile	Tyr	Met	5.14
BF-841 cl1	Z-XIVhk2-	C	G	C	A	T	C	T	C	G	Gly	Gln	Phe	Leu	Met	5.19
KU2	Z-XIX	T	C	G	T	A	T	A	T	G	Ala	Asp	Ile	Tyr	Met	5.14

* The reference sequence data of HM-1:IMSS cl6 (XM_650873) strain is represented as numbers at variable positions in the hexokinase-2 gene. Polymorphic sites are highlighted in gray.

**Table IV.**
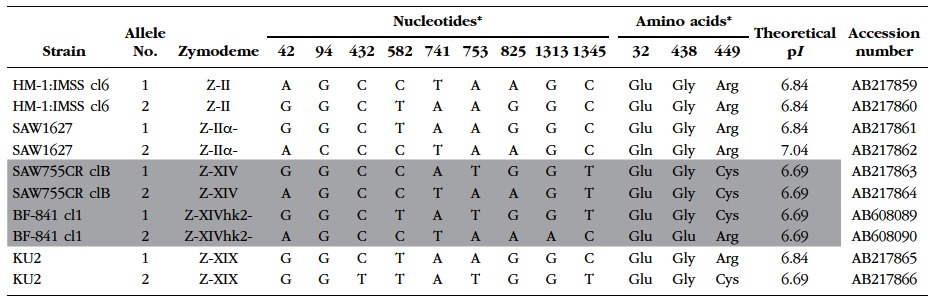
Polymorphic sites of glucose phosphate isomerase (GPI) in BF-841 cl1 and reference strains possessing four different types of zymodemes of *Entamoeba histolytica*.

			Nucleotides*									Amino acids*				
Strain	Allele No.	Zymodeme	42	94	432	582	741	753	825	1313	1345	32	438	449	Theoretical p*I*	Accession number
HM-1:IMSS cl6	1	Z-II	A	G	C	C	T	A	A	G	C	Glu	Gly	Arg	6.84	AB217859
HM-1:IMSS cl6	2	Z-II	G	G	C	T	A	A	G	G	C	Glu	Gly	Arg	6.84	AB217860
SAW1627	1	Z-IIa-	G	G	C	T	A	A	G	G	C	Glu	Gly	Arg	6.84	AB217861
SAW1627	2	Z-IIa-	A	C	C	C	T	A	A	G	C	Gln	Gly	Arg	7.04	AB217862
SAW755CR clB	1	Z-XIV	G	G	C	C	A	T	G	G	T	Glu	Gly	Cys	6.69	AB217863
SAW755CR clB	2	Z-XIV	A	G	C	C	T	A	A	G	T	Glu	Gly	Cys	6.69	AB217864
BF-841 cl1	1	Z-XIVhk2-	G	G	C	T	A	T	G	G	T	Glu	Gly	Cys	6.69	AB608089
BF-841 cl1	2	Z-XIVhk2-	A	G	C	C	T	A	A	A	C	Glu	Glu	Arg	6.69	AB608090
KU2	1	Z-XIX	G	G	C	T	A	A	G	G	C	Glu	Gly	Arg	6.84	AB217865
KU2	2	Z-XIX	G	G	T	T	A	T	G	G	T	Glu	Gly	Cys	6.69	AB217866

* The reference sequence data of HM-1:IMSS cl6 (AB217859) strain of *E. histolytica* is represented as numbers at variable positions in the GPI gene. The rows for SAW755CR clB and BF-841 cl1 present approximate zymodemes are highlighted in gray.

In the *PGM* gene nucleotide base sequence of BF-841 cl1, one base substitution was present, as compared to that of HM-1:IMSS (XM_651929)-adenine at position 963 was substituted with guanine; no substitution was present in the amino acid sequence. However, the PGM sequence of BF-841 cl1 (AB608091) was identical to those of SFL-3 (Y14444), SAW1627, SAW755CR clB, KU2, and KU27. The *ME* gene nucleotide base sequences of BF-841 cl1 (AB608098), SAW1627, SAW755CR clB, and KU2 were identical to that of HM-1: IMSS (NW_001914864).

**Fig. 3.**
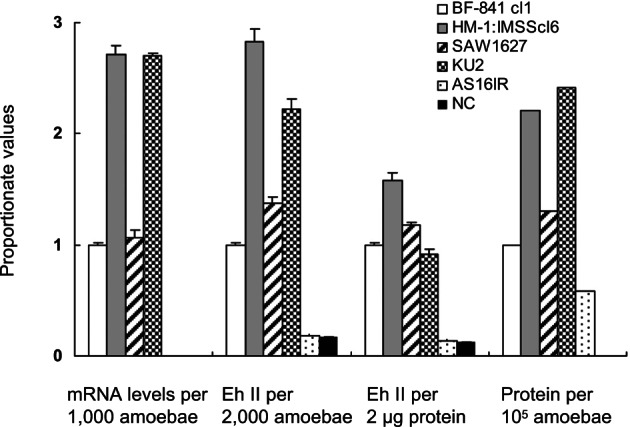
Proportionate values among four examined strains of *Entamoeba histolytica* for the levels of the following: mRNA of the galactose/ *N*-acetyl-d-galactosamine-inhibitable lectin gene (adhesin) per a fixed number of amoebae, captured antigen of adhesin per a fixed amount of amoebae protein, and protein content per a fixed number of amoebae.

The proportionate values for mRNA quantity in HM-1: IMSS cl6 and KU2 were shown to be high, *i.e.* 2.71 and 2.70; similar values were observed for SAW1627 (1.06) and BF-841 cl1 (1.0). Similarly, a significant difference was observed among the values of captured-antigen levels of adhesin per 2,000 amoebae, but such a difference was not observed among the values per 2 µg protein of freeze-thawed lysates of amoebae for the four strains, as shown in Fig. 3. This pattern, where a difference was observed between the proportionate values that were based on cell numbers and those based on protein contents of the four strains, was also seen with the values for mRNA level. Hence, it was proposed that the variation is related in part to differences between the cell sizes of the different strains of amoebae. In fact, the sizes of BF-841 cl1 and SAW1627 were considerably smaller than those of HM-1:IMSS cl6 and KU2 (data not shown).

The ability of BF-841 cl1 to form amoebic liver abscesses in Syrian hamster was confirmed; the weights of the abscesses reached ~ 3,000-4,000 mg six days after inoculation with amoebae, which were in the same range as the abscesses formed by the reference virulent strain (HM-1:IMSS cl6, data not shown).

## Discussion

In the present study, a novel variation in the electrophoretic mobility of HXK-2 of *E. histolytica* was found in an isolate (BF-841 cl1) that originated in Africa. Further investigation revealed that BF-841 cl1 had four amino acid substitutions in its HXK-2 gene, as compared with known reference strains of *E. histolytica* and a novel genotype of SREHP with the seldom seen triple PCR-amplified DNA bands (Haghighi *et al.*, 2003).

The existence of interspecies differences in the electrophoretic mobility of isoenzymes of HXK-1 and HXK-2 between tissue-invasive *E. histolytica* and noninvasive *E. dispar* is well documented (Sargeaunt, 1988). Ramón *et al.*, (1974) studied monosaccharide transport in *E. histolytica* and proved the existence of a specific transport system for glucose, which is a major substrate for HXK. In another study, Kroschewski *et al.* (2000) reported the differences between the characteristics of recombinant HXK-1 and HXK-2 from *E. histolytica* strain SFL-3; for example, glucose and mannose are both readily phosphorylated by HXK-1, mannose is phosphorylated at a much lower rate by HXK-2 than by HXK-1, and HXK-1 is more sensitive to inhibition by AMP and ADP than is HEX-2. Irrespective of the differences between the gene nucleotide and amino acid sequences of HXK-2 from BF-841 cl1 and other strains of *E. histolytica*, and among the six strains used in this study [four strains of *E. histolytica* and one each of *E. histolytica*-like variants (*E. nuttalli*) and *E. dispar*)], the reactions of HXK-1 and HXK-2 were similar in our IEF analyses (Fig. 1). All results were consistent with the characteristic differences between recombinant HXK-1 and HXK-2 constructed from the HXK gene nucleotide alignment of SFL-3, as described by Kroschewski *et al.* (2000). In the protein structural model of HXK-2 from BF-841 cl1 that was theoretically constructed by SWISS-MODEL (http://swissmodel. expasy.org; Arnold *et al.*, 2006; Kiefer *et al.*, 2009) based on the structures of highly homologous HXK from Schistosoma mansoni (PDB ID: 1BDG) and HXK PII of Saccharomyces cerevisiae (PDB ID: 1IG8), the active sites for ATP and glucose exhibited very similar conformations in the HXK-2 of both BF-841 cl1 and HM-1:IMSS cl6.

The mannokinase activities of HXK-1 and HXK-2 were measured to be of the same level when using the reaction mixture for glucokinase (shown in Fig. 1: IIc) because the modified M6P assay system required a considerably longer time for reaction completion than did the M6P-specific assay system. Thus, it is possible that the mannokinase activity of HXK-1 was more affected and considerably decreased by the ADP that was produced by ATP dephosphorylation in the substrate solution of the modified M6P assay system as compared to in the common M6P-specific assay system. Regarding the mannokinase activity of HXK-2, ADP did not seem to substantially affect the activity.

These results may suggest that the HXK-2 genes of these *Entamoeba* spp. have a common compensatory role for HXK-1 in ensuring adequate phosphorylation of glucose and mannose in the event that HXK-1 is inhibited by ADP or AMP.

A complete understanding of the variation of HXK-2 found in BF-841 cl1 could not be achieved in the present study, but the findings of this study suggest the interesting possibility that the source of variation was the initial enzyme of glycolysis, involving the energy metabolism of pathogenic *E. histolytica*.

Some unknown, latent minor variants of *E. histolytica* are predicted to exist. However, to date, the established axenic strains of *E. histolytica* seem to be of a closely similar heritable nature, as if they were axenized by a selective culture. Further isolation of unique variants that may not be adaptable to present axenic culture systems may require development of a new axenic culture system that is adaptable and applicable for a broad range of lumen-parasitic *Entamoeba* species.
